# A cost-effective colourimetric assay for quantifying hydrogen peroxide in honey

**DOI:** 10.1099/acmi.0.000065

**Published:** 2019-10-21

**Authors:** D. M. Lehmann, K. Krishnakumar, M. A. Batres, A. Hakola-Parry, N. Cokcetin, E. Harry, D. A. Carter

**Affiliations:** ^1^​ Immediate Office, National Health and Environmental Effects Research Laboratory (NHEERL), US - Environmental Protection Agency, Research Triangle Park, NC, USA; ^2^​ School of Life and Environmental Sciences, University of Sydney, NSW 2006, Australia; ^3^​ Oak Ridge Associated Universities, Raleigh, NC, USA; ^4^​ ithree Institute, University of Technology Sydney, NSW 2007, Australia; ^5^​ Marie Bashir Institute, University of Sydney, NSW 2006, Australia

**Keywords:** honey, hydrogen peroxide, horse radish peroxidase, glucose oxidase, *o*-dianisidine, colourimetric assay

## Abstract

Honey is a natural product with many beneficial properties including antimicrobial action. Production of hydrogen peroxide (H_2_O_2_) in diluted honey is central to this action. Here, we describe an optimized method for measuring levels of H_2_O_2_ in honey. This method is based on established methods, with the level of dilution, the time between dilution and reading the assay, and aeration of the samples during the assay identified as critical points for ensuring reliability and reproducibility. The method is cost-effective and easy to perform using common laboratory equipment. Using this method, we quantified the hydrogen peroxide content of five different, unprocessed polyfloral honeys collected in NC, USA. Our results show that H_2_O_2_ production by these honeys varies greatly, with some samples producing negligible levels of H_2_O_2_. We assessed the effect of colour on the assay by measuring the recovery of spiked H_2_O_2_ from light and dark honey and from serially diluted dark corn syrup, and found the amount of H_2_O_2_ that could be detected was lower in dark corn syrup and darker honey samples.

## Introduction

Honey is composed of approximately 83 % sugar, primarily glucose and fructose, and about 17 % water with an average pH of 3.9 [1]. However, it is more than just a supersaturated sugar solution; honey is a complex mixture composed of sugars, proteins, amino and organic acids, flavonoids, polyphenols, carotenoid-like substances, Maillard reaction products [i.e. 5-hydroxymethyl(fural)], vitamins, minerals and water [2]. The actual composition of honey varies and depends on factors including nectar source, pollen content, foraging time of year and other elements of the environment [3]. Due to its unique properties, honey has been utilized as a food source for humans and also has proven useful as a topical treatment for wounds and bacterial infections [4].

Laboratory-based research and a limited number of clinical studies have demonstrated that honey possesses broad-spectrum antimicrobial properties against bacteria, fungi, viral and mycobacterial pathogens [5–14], with maximal effects observed in fresh, unheated honey [15–18]. All honeys possess intrinsic characteristics that, in combination, can inhibit microbial growth and survival. The antimicrobial properties of some honeys are augmented by other compounds introduced by the bees themselves or through their diet, including lysozyme, flavonoids and polyphenols [2]. In addition, phytochemically derived methylglyoxal (MGO) and the antimicrobial peptide bee defensin-1 (i.e. royalisin) were determined to be novel mechanisms of antibacterial action in Manuka honey and RS honey, respectively [6, 19, 20]. Antimicrobial effects also stem from hydrogen peroxide (H_2_O_2_) in honey, which is produced by glucose oxidase, an enzyme introduced into nectar by worker bees [21]. In the presence of certain metals, H_2_O_2_ decomposes to form reactive oxygen species, which drive lipid peroxidation, ultimately destroying microbes [22].

Glucose oxidase is inactive in fully ripened honey [23]. However, when honey is diluted, glucose oxidase converts β-d-glucose into H_2_O_2_ and d-Gluconic acid [6]. The amount of H_2_O_2_ produced is dependent on the type of honey, honey age and storage conditions (i.e. light exposure, temperature and filtration), honey dilution rate, and length of time since dilution [18, 24]. H_2_O_2_ accumulation is greatest in honey samples diluted to 30–50 % strength; due to the low affinity of glucose oxidase for glucose, accumulation decreases when honey is diluted below 30 % [23].

Given the high level of interest in the use of honey for wound healing [5, 6, 10, 25–28], characterizing the capacity of honey to produce H_2_O_2_ is of great interest to medical practitioners, complementary and alternative medicine communities, food chemists and beekeepers. The AmplexRed assay is a reliable test that has been used in a number of studies, however it is relatively expensive and is more suited to medium-to-large throughput studies. A colourimetric assay using horseradish peroxidase (HRP) to catalyse the oxidation of colourless *o*-dianisidine by H_2_O_2_ to a coloured product has been in use for a number of years [6, 18, 29–32], but detailed optimized methodology is lacking, requiring researchers to perform considerable trouble-shooting. Below, we provide this as an optimized, easy-to-perform and cost-effective protocol for the quantification of H_2_O_2_ in honey. We demonstrate the utility of this method by examining the H_2_O_2_ production capacity of five different polyfloral honeys collected in the NC, USA. In addition, we show how the capacity to detect H_2_O_2_ can be quenched in darker coloured honey and corn syrup samples.

## Methods

### Optimized H_2_O_2_ assay

**Fig. 1.**
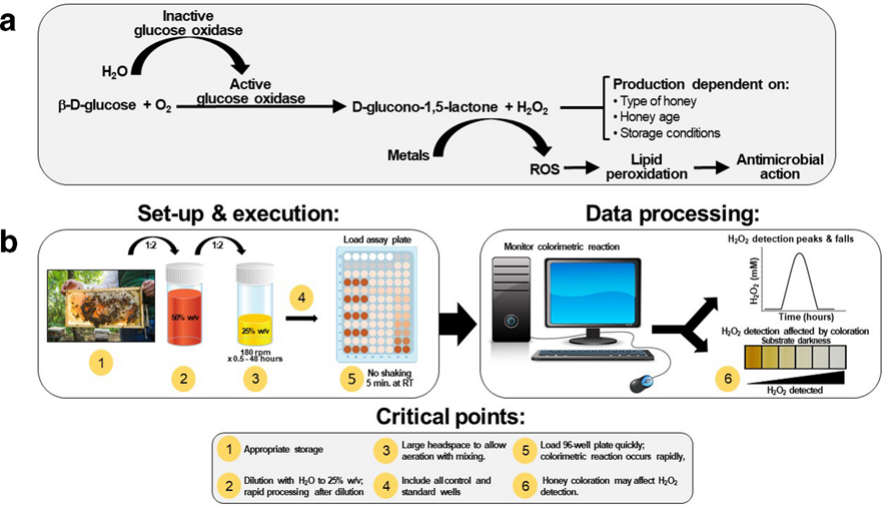
H_2_O_2_ production mechanism and methodological approach. (a) H_2_O_2_ is produced in diluted honey by a reaction involving β-d-glucose, O_2_ and activated glucose oxidase. Production of H_2_O_2_ is influenced by the indicated parameters. (b) H_2_O_2_ production can be easily quantified using the optimized colourimetric assay described here provided attention is paid to by several critical points in the set-up and execution of the assay. ROS=reactive oxygen species.

We used a previously developed colourimetric assay to determine the concentration of hydrogen peroxide in honey [6, 18] with modifications that enabled significant improvements in reproducibility and reliability. This assay is based on the fact that upon dilution of honey with water, H_2_O_2_ production by glucose oxidase is activated, and this is detected by the oxidation of colourless *o*-dianisidine reagent catalysed by HRP, resulting in the formation of a coloured product that is detected spectrophotometrically (Fig. 1).

### Preparation of reagent solutions

In total, 1 M sodium phosphate monohydrate, anhydrous, monobasic, was prepared by dissolving 6.9 g NaH_2_PO_4_-H_2_O in a total volume of 50 ml distilled water. Then, 1 M disodium phosphate anhydrous, dibasic was prepared by dissolving 7.1 g Na_2_HPO_4_ in a total volume of 50 ml distilled water with gentle heating until all solid was visibly dissolved. Next, 10 mM sodium phosphate buffer, pH 6.5, was prepared by adding 3.15 ml Na_2_HPO_4_ and 6.85 ml NaH_2_PO_4_-H_2_O to 990 ml sterile, distilled water and adjusting to pH 6.5 with Na_2_HPO_4_ (if too acidic) or NaH_2_PO_4_-H_2_O (if too basic). All buffers were filter sterilized using a 0.45 µm filter and stored at 4 ˚C.

A 5 mg ml^−1^ stock solution of *o*-dianisidine was prepared by adding 20 mg of *o*-dianisidine (Sigma Aldrich, Cat. No. D9143) to 4 ml 95 % ethanol with gentle mixing until all *o*-dianisidine dissolved. On the day of experimentation, 250 µl of this was added to 1 ml of the 10 mM sodium phosphate buffer (pH 6.5) to make a 1 mg ml^−1^ working solution.

Then, 10 mg ml^−1^ HRP, type II, made fresh on the day of experimentation, was prepared by adding 10 mg HRP, type II (Sigma Aldrich, Cat. No. P8250-25KU) to 1 ml 10 mM sodium phosphate buffer with gentle mixing until the HRP dissolved. Next, 2 mg ml^−1^ catalase solution, also made fresh on the day of experimentation, was made by adding 10 mg catalase to 5 ml of 10 mM sodium phosphate buffer with gentle mixing until fully dissolved. Catalase blank solution consisted of 10 mM sodium phosphate buffer only. Then, 6M sulfuric acid was prepared by slowly adding 67 ml of 18 M H_2_SO_4_ to 50 ml distilled water and adjusting to a final volume of 200 ml with distilled water.

HRP reagent mixture solution was prepared by combining 1 ml of 1 mg ml^−1^ *o*-dianisidine working solution and 40 µl of 10 mg ml^−1^ HRP stock with 18.96 ml of the 10 mM sodium phosphate buffer (this makes enough for 148 wells). This was left at room temperature until used, and any remaining solution was discarded once the assay was completed.

### Preparation of honey samples and H_2_O_2_ standards

All of the following preparation was done on the day of experimentation and was performed under subdued lightning.

To prepare the honey samples, 4 g of each honey was added to 4 ml sterilized dH_2_O pre-warmed to 37 °C and incubated at 35 °C protected from light on an orbital shaker (~180 r.p.m.) for 20 min to aid mixing. The resulting 50 % (w/v) stock solutions were filter sterilized through a 0.22 µm pore filter (Millex). Aliquots (2.5 ml) of the sterilized honey were then transferred to 28 ml McCartney bottles and further diluted to 25 % (w/v) using either sterile deionized water, catalase solution or catalase blank solution. The McCartney bottles were protected from light using aluminum foil and incubated at 35 °C in an orbital shaking incubator at 180 r.p.m. for various times to enable a time-course (initially from 0.5 to 48 h and subsequently from 2 to 18 h). Agitating the diluted honey with this large headspace volume enabled thorough aeration of the sample, which proved critical for maximal and reliable H_2_O_2_ production. When preparing multiple honey samples, dilutions were done at the same time for all samples.

To prepare the H_2_O_2_ standards, 10 ml of 8.8 mM H_2_O_2_ was prepared from 0.88 M H_2_O_2_ stock using sterile dH_2_O, and this was further diluted to make 1 ml of 2.2 mM H_2_O_2_. From this, 500 µl was aliquoted into an amber 1.5 ml tubes, and 250 µl of 10 mM sodium phosphate buffer was aliquoted into an additional ten amber tubes. The H_2_O_2_ solution was then serially diluted from the first tube and across the remaining ten tubes at a 1 : 1 ratio to produce 2200, 1100, 550, 275, 137.5, 68.8, 34.4, 17.2, 8.6, 4.3, 2.1 µM H_2_O_2_ standards. All tubes were vortexed well between dilutions to mix thoroughly. A 0 µM H_2_O_2_ standard was prepared by combining 250 µl of 10 mM sodium phosphate buffer with 250 µl sterile dH_2_O. All H_2_O_2_ standards were used within 2 h of preparation.

**Fig. 2.**
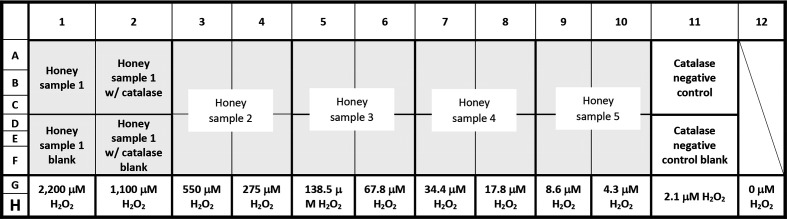
The 96-well microplate lay-out for quantification of hydrogen peroxide in honey samples. Each honey sample requires 12 wells divided into quadrants with three replicates in each quadrant. Quadrants consist of honey alone and three honey-containing controls. This scheme allows for up to five honey samples and catalase controls to be assayed (in triplicate) on a single plate. The diagonal line in column 12 indicates empty assay plate wells.

Working in subdued light, 96-well flat-bottomed microtitre assay plate(s) were loaded in the following manner (see also Fig. 2):

### Standards

*H_2_O_2_ standards –* 40 µl of the serially diluted H_2_O_2_ standards were aliquoted into wells G1-12 and H1 – 12, followed by 135 µl of HRP reagent mixture solution.

*Catalase negative control* **–** 20 µl of 550 µM H_2_O_2_ standard and 20 µl of catalase solution were added to wells A11, B11 and C11, followed by 135 µl of HRP reagent mixture solution.

*Catalase negative control blank* **–** 20 µl of 550 µM H_2_O_2_ standard and 20 µl of 10 mM sodium phosphate buffer were added to wells D11, E11 and F11, followed by 135 µl of HRP reagent mixture solution.

### Honey samples

*Honey sample no. 1 test* – 40 µl of the first honey sample (diluted 25 % w/v in dH_2_O) was added to wells A1, B1 and C1, followed by 135 µl of HRP reagent mixture solution.

*Honey sample no. 1 blank* **–** 40 µl of the first honey sample (diluted 25 % w/v in dH_2_O) was added to wells D1, E1 and F1, followed by 135 µl of 10 mM sodium phosphate buffer.

*Honey sample no. 1 with catalase* **–** 40 µl of the first honey sample (diluted 25 % w/v with catalase solution) was added to wells A2, B2 and C2, followed by 135 µl of HRP reagent mixture solution.

*Honey sample no. 1 with catalase blank* **–** 40 µl of the first honey sample (diluted 25 % w/v with catalase blank solution) was added to wells D2, E2 and F2, followed by 135 µl of 10 mM sodium phosphate buffer.

The above was repeated for each additional honey sample, allowing a total of five samples to be tested per plate. Once fully loaded, the plate was covered with a lid and tinfoil to protect from light, tapped gently on the side to mix and incubated for 5 min at room temperature (no shaking). To stop the reaction, 120 µl 6M H_2_SO_4_ was then added to all wells and mixed by gently tapping the side of the plate. The foil and plate lid were then removed and absorbance at OD_560_ was read using a spectrophotometer. This assay produces a quantifiable H_2_O_2_ range of 0 to 550 µM.

### Data analysis

*Plotting the standard curve* – Blank-corrected H_2_O_2_ standards were calculated by subtracting the mean absorbance of the 0 µM H_2_O_2_ standard from the mean absorbance value for each H_2_O_2_ standard. These data were then used to generate a standard curve in GraphPad Prism, with concentration on the *x*-axis and mean absorbance on the *y*-axis. As absorbance increases until H_2_O_2_ reaches 550 µM and then declines, the first portion of the graph (0–550 µM) was used to fit a linear trend line with the equation *y*=*mx*+*b*, where *m*=slope of the line and *b*=the *y* intercept.

**Fig. 3.**
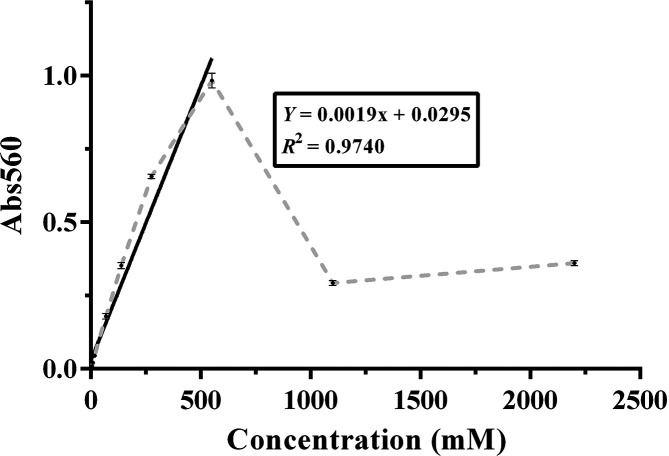
Typical standard curve for quantification of H_2_O_2_. Mean absorbance values were plotted for blank-corrected H_2_O_2_ standards ranging from 0 to 2200 µM (grey broken line). The solid black line indicates the linear portion of the curve used for calculations. Data shown *n*=3, ±sd. For some points, error bars are very small (0.1–0.25) and therefore difficult to see.

*Determining the concentration of H_2_O_2_ in the honey samples* – The intensity of the coloured reaction produced in the honey samples is directly related to their level of H_2_O_2_ production. The latter is therefore calculated by comparing the absorbance value with the standards and reading the concentration from the standard curve (Fig. 3). Since honey is a coloured product that can vary considerably according to floral source and age, it is important to subtract the honey blank (no HRP reagent mixture solution) from the honey test. Catalase-treated honey samples were included to confirm that the observed values were in fact the result of H_2_O_2_ production; if this was the case the absorbance of the honey sample with catalase should be the same as that of the same honey sample without catalase. As the assay is critically dependent on incubation time and temperature, separate standard curves should be calculated from each assay plate to ensure standardization across tests.

### Application of the assay to freshly collected honey samples

North Carolina beekeepers collected honey directly from honeycomb into sterile, tightly capped, black 50 ml polypropylene tubes. Colonies were not receiving supplemental feed during the time of honey collection. Within 4 h of field collection, honey samples were assigned a sample number, passed through a 100 µm filter to remove debris, partitioned into 2.5 g single-use aliquots and transferred to cold storage at 4 ˚C protected from light. The pH of each honey sample was assessed following dilution to 10 % (w/v) in Milli-Q water. Honey moisture content was determined using a Misco BKPR-1 (Solon, OH, USA) refractometer and following the manufacturer’s instructions. Testing was undertaken as outlined above.

### Assessment of the effect of honey colour on H_2_O_2_ detection

Honey samples can vary considerably in colour, and this may affect the assay readings. To test this, clear corn syrup (CCS; a sugar solution with no glucose oxidase), dark corn syrup (DCS; CCS with added caramel colour and molasses), buckwheat honey (which is very dark in colour) and clover honey (which is very pale) were heated at 70 ˚C for 1 h to eliminate glucose oxidase and catalase activities [33]. Samples were then diluted (to 50 % w/v with water for CCS and the honey samples, to 50 % w/v with water for DCS and then in doubling dilutions to 1.56 % w/v in CCS), and 250 µl was added to the microwell plates and spiked with and equal volume of 1 mM H_2_O_2_ to achieve a final concentration of 25 % CCS, DCS or honey and 500 µM H_2_O_2_. H_2_O_2_ levels were assayed immediately according to the optimized protocol, with incubation for 5 min.

## Results and discussion

### Optimization of the HRP–*o*-dianisidine colourimetric method for detecting H_2_O_2_ production in honey

Hydrogen peroxide, well known as an antimicrobial agent, is responsible for the antimicrobial activity of the majority of honey types. Peroxide activity can be determined via biological testing that compares killing of a test strain of bacterial pathogen *Staphylococcus aureus* by honey in the presence and absence of catalase using a well diffusion assay [31]. If all activity is abolished by catalase it is assumed to be due to H_2_O_2_, and this can be quantified by comparison to established phenol standards. While on the surface a simple test, the well diffusion assay is difficult to standardize, highly dependent on culture conditions including the quality and quantity of agar, quality of the tester strain, and incubation temperature and time, requiring considerable time, a skilled operator, a laboratory certified for handling bacterial pathogens and a high level of attention to detail. In addition, the biological assay is an indirect measure of H_2_O_2_ as an assessment of toxicity to bacterial cells. Chemical tests could by-pass these issues, enabling a rapid, high-throughput assay that is simple and cost-effective to perform.

**Fig. 4.**
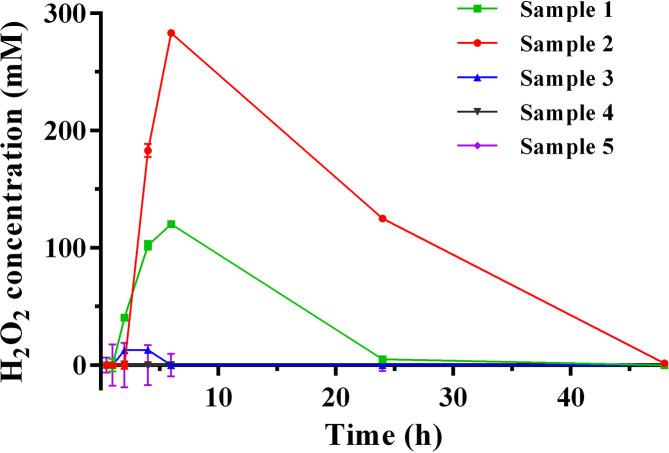
Time course for H_2_O_2_ production in five different freshly collected honey samples. Following dilution, the concentration of H_2_O_2_ in honey (25 % w/v) was assayed at various time points. Although collected at the same time and from the same region, the different samples varied substantially in H_2_O_2_ production levels. *n*=3, error bars show ±sd. Error bars ranged from 0.4 to 19 and were particularly small at the higher H_2_O_2_ concentrations, making these difficult to see.

At the outset of this study, we found the HRP–*o*-dianisidine colourimetric method to be variable and difficult to standardize. The following parameters were identified as being critical to the successful deployment of this method:

1. Honey dilution – H_2_O_2_ accumulation is known to be highest when honey is diluted to 30–50% strength; below 30 % the low-affinity glucose oxidase becomes limiting, and above 50 % there is too little free water for H_2_O_2_ production [23]. However, here (and in previous studies) 25 % honey was used, which provided an optimal trade-off between maximal H_2_O_2_ production and simplicity of the assay.
2. Dilution time – The kinetics of H_2_O_2_ production and degradation varied among honey samples such that the time of incubation for maximum production occurred anywhere between 3–6 h, depending on the sample (Fig. 4). Generally, samples with higher levels of H_2_O_2_ had later peaks in production, and the overall kinetics are in good agreement with other published reports [21, 24, 34]. It is recommended that at least two timepoints (4 and 6 h) be tested for each sample to maximize the chance of seeing peak production.
3. Aeration – Incubation with a large headspace volume was found to be critical for maximal H_2_O_2_ production. Here we found using 5 ml honey in 28 ml McCartney bottles with shaking at 180 r.p.m. provided ideal aeration and greatly improved the assay.
4. Rapid workflow – As the assay components degrade quickly over time it is essential to work quickly, ensuring all reagents are made just prior to use and that the plates are loaded in the shortest time possible. We recommend doing no more than two plates at one time.

This optimized protocol is expected to be broadly applicable based on published ranges for H_2_O_2_ in honey [6, 18, 21, 24, 35]. Our protocol provides sufficient detail to allow adoption by new users. If an investigator needs to customize the assay, the protocol can be easily modified using the detailed reagent formulae and the rationale for assay design and execution provided here.

### Application of the optimized method for quantification of H_2_O_2_ in honey samples

H_2_O_2_ production capacity was evaluated for five unprocessed polyfloral honey samples using our optimized method. All samples had typical pH and moisture content (Table 1) [2]. Each honey sample was diluted to a final concentration of 25 % (w/v) and evaluated for production of H_2_O_2_ over a multipoint time course (Fig. 4). For honey samples 1 and 2, H_2_O_2_ production peaked 6 h post-dilution to 120.4±3.0 µM and 283.2±0.4 µM, respectively, and returned to zero by 48 h (Table 1 and Fig. 4). Sample 3 produced low (13.2±1.2 µM), but detectible H_2_O_2_ levels. H_2_O_2_ was not detected in samples 4 and 5. In all instances, assay responses were eliminated by incubation with catalase, indicating specificity of the assay for H_2_O_2_.

**Table 1.** Moisture content, pH, and H_2_O_2_ production of honey samples collected in NC, USA

Honey sample	pH		Moisture content (%)		Maximum H_2_O_2_ production (μM)	
	Mean	sd	Mean	sd	Mean	sd
1*	4.1	0.1	17.3	0.6	120.4	3.0
2*	4.3	0.2	18.8	0.6	283.2	0.4
**3**	3.8	0.1	16.8	0.4	13.2	1.2
**4**	4.6	0.2	19.3	0.4	0	0.7
**5**	5.0	0.2	16.4	0.6	0	9.6

*Peak H_2_O_2_ production occurred at 6 h for samples 1 and 2 and 4 h for sample 3. Mean derived from three replicates. sd=standard deviation.

Differences in H_2_O_2_ accumulation capacity of different honeys are well-known [18, 35] and have been attributed to floral source [9, 25, 36], foraging time of year, honey age, storage conditions [37] and processing [18]. Polyfloral honeys were used in this work, and all were collected during a 1 week period in the same general geographical area before being stored and processed under the same conditions. Given that glucose oxidase is derived from bees one might expect similar levels in minimally processed honey. This high level of variability may reflect differences in bee and hive health, or environmental conditions such as temperature and humidity. Other components present in the honey might also augment or suppress glucose oxidase activity; for example, catalase can be introduced into honey with pollen grains [21, 38, 39]. As noted below, colour may affect the H_2_O_2_ readout, however all of the North Carolina honeys used for this study were of a similar hue (Pfund colour range >35 to 50 mm).

### Honey colour can influence the capacity to detect H_2_O_2_ by the HRP–*o*-dianisidine assay

**Fig. 5.**
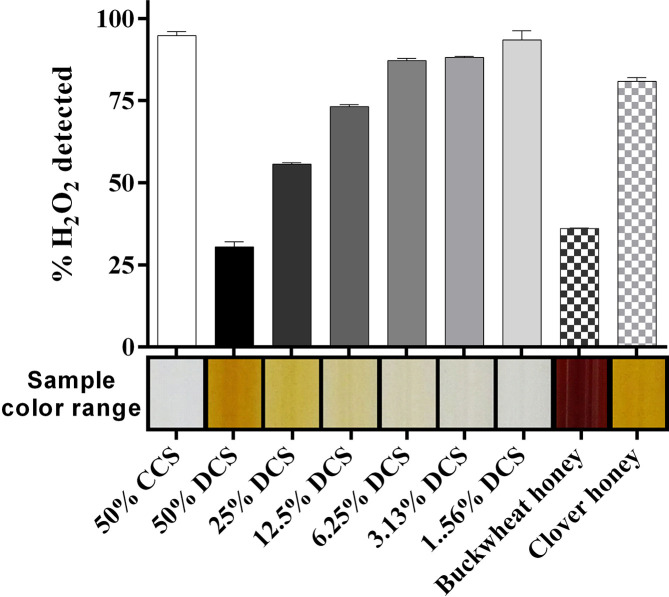
Sample colour and H_2_O_2_ detection. Clear corn syrup (CCS), dark corn syrup (DCS), and dark- and light-coloured store-bought honey samples were heated to eliminate glucose oxidase and catalase, diluted and spiked to a final concentration of 500 µM H_2_O_2_. H_2_O_2_ levels were assayed immediately after spiking with H_2_O_2_, according to our optimized protocol. The coloured bar on the horizontal axis shows the approximate colour of each sample. DCS reduced H_2_O_2_ detection in a dose-dependent manner, and substantial quenching was also seen in the dark buckwheat honey sample. *n*=3, error bars show ±sd, which ranged from 0.22 to 2.8.

Honey samples vary considerably in colour due to the presence of phenolics, flavonoids and maillard reaction products that are produced when sugars and other components age. To test the influence of colour we spiked heat-treated clear corn syrup (CCS) and dark corn syrup (DCS; from 1.56–50 % w/v) and a light and dark honey sample with a known amount of H_2_O_2_ and looked for recovery in the assay. The lightest matrix tested, 50 % (w/v) CCS, was the only vehicle where the H_2_O_2_ was fully recovered (Fig. 5). In contrast, 50 % (w/v) DCS reduced detectable H_2_O_2_ to 33.9±0.4 % of the original level. Diluting the DCS with CCS increased the detection of H_2_O_2_ in a dose-dependent manner. Similarly, buckwheat honey, which is very dark, yielded only 36.1±0.2 % of the spiked H_2_O_2_ whereas 80.9±1.1 % was detected in the much paler clover honey (Fig. 5). Our data are the first to suggest that H_2_O_2_ levels may be underestimated when using this colourimetric assay with darker honeys.

### Concluding comments

This report details an optimized procedure for quantifying H_2_O_2_ in honey and presents a strategy for evaluating the H_2_O_2_ production capacity of honeys. Prior to this work, a detailed protocol and strategy for quantification of H_2_O_2_ in honey based on the oxidation of *o*-dianisidine was not available. Our optimized protocol is expected to be broadly applicable based on published ranges for H_2_O_2_ in honey. The assay is cost-effective and easily performed using common laboratory equipment. We highlight critical steps in the protocol that enable maximal and reliable H_2_O_2_ production. Additional research is required to better understand the potential impact of colour and other honey components on H_2_O_2_ quantification. As various factors within honey may act to suppress or augment H_2_O_2_ levels or interfere with their detection, our work suggests that this method is best-suited for studies tracking changes in H_2_O_2_ production capacity over time or in response to processing and storage conditions in a single sample and may not be so well-suited for comparing H_2_O_2_ levels in honey samples, particularly if these are of significantly different hues.

### Disclaimer

This article has been reviewed by the U.S. Environmental Protection Agency and approved for publication. Approval does not signify that the contents necessarily reflect the views and policies of the Agency or of the US Federal Government, nor does the mention of trade names or commercial products constitute endorsement or recommendations for use of those products. The authors report no financial or other conflicts of interest. The authors alone are responsible for the content and writing of this article.

## References

[1] White JW, Doner LW (1980). Honey composition and properties: Beekeeping in the United States. Agriculture Handbook.

[2] Machado De-Melo AA, Almeida-Muradian LBde, Sancho MT, Pascual-Maté A (2018). Composition and properties of *Apis mellifera* honey: a review. J Apic Res.

[3] Alvarez-Suarez JM, Gasparrini M, Forbes-Hernández TY, Mazzoni L, Giampieri F (2014). The composition and biological activity of honey: a focus on manuka honey. Foods.

[4] Ball DW (2007). The chemical composition of honey. J Chem Educ.

[5] Israili ZH (2014). Antimicrobial properties of honey. Am J Ther.

[6] Kwakman PHS, te Velde AA, de Boer L, Speijer D, Vandenbroucke-Grauls CMJE (2010). How honey kills bacteria. Faseb J.

[7] Aljadi AM, Yusoff KM (2003). Isolation and identification of phenolic acids in Malaysian honey with antibacterial properties. Turk J Med Sci.

[8] Henriques A, Jackson S, Cooper R, Burton N (2006). Free radical production and quenching in honeys with wound healing potential. J Antimicrob Chemother.

[9] Brudzynski K (2006). Effect of hydrogen peroxide on antibacterial activities of Canadian honeys. Can J Microbiol.

[10] Bogdanov S (1997). Nature and origin of the antibacterial substances in honey. LWT - Food Sci Technol.

[11] Wahdan HA (1998). Causes of the antimicrobial activity of honey. Infection.

[12] Kassim M, Achoui M, Mansor M, Yusoff KM (2010). The inhibitory effects of Gelam honey and its extracts on nitric oxide and prostaglandin E(2) in inflammatory tissues. Fitoterapia.

[13] Weston RJ (2000). The contribution of catalase and other natural products to the antibacterial activity of honey: a review. Food Chem.

[14] Molan PC (2006). The evidence supporting the use of honey as a wound dressing. Int J Low Extrem Wounds.

[15] Bogdanov S (2016). Honey as nutrient and functional food: a review. Bee Product Science.

[16] White JW, Subers MH (1964). Studies on honey inhibine. 3. Effect of heat. J Apic Res.

[17] White JW, Subers MH (1964). Studies on honey inhibine. 4. destruction of the peroxide accumulation system by light. J Food Sci.

[18] Chen C, Campbell LT, Blair SE, Carter DA (2012). The effect of standard heat and filtration processing procedures on antimicrobial activity and hydrogen peroxide levels in honey. Front Microbiol.

[19] Adams CJ, Boult CH, Deadman BJ, Farr JM, Grainger MNC (2008). Isolation by HPLC and characterisation of the bioactive fraction of New Zealand manuka (*Leptospermum scoparium*) honey. Carbohydr Res.

[20] Mavric E, Wittmann S, Barth G, Henle T (2008). Identification and quantification of methylglyoxal as the dominant antibacterial constituent of manuka (*Leptospermum scoparium*) honeys from New Zealand. Mol Nutr Food Res.

[21] White JW, Subers MH, Schepartz AI (1963). The identification of inhibine, the antibacterial factor in honey, as hydrogen peroxide and its origin in a honey glucose-oxidase system. Biochim Biophys Acta.

[22] Juven BJ, Pierson MD (1996). Antibacterial effects of hydrogen peroxide and methods for its detection and quantitation. J Food Prot.

[23] Schepartz AI, Subers MH (1964). The glucose oxidase of honey. I. Purification and some general properties of the enzyme. Biochim Biophys Acta.

[24] Bang LM, Buntting C, Molan P (2003). The effect of dilution on the rate of hydrogen peroxide production in honey and its implications for wound healing. J Altern Complement Med.

[25] Kwakman PHS, Te Velde AA, de Boer L, Vandenbroucke-Grauls CMJE, Zaat SAJ (2011). Two major medicinal honeys have different mechanisms of bactericidal activity. PLoS One.

[26] Bernstein RC (2013). The scientific evidence validationg the use of honey as a medicinal agent. The Science Journal of the Lander College of Arts and Sciences.

[27] Afroz R, Tanvir EM, Zheng W, Little PJ (2016). Molecular pharmacology of honey. Journal of Clinical & Experimental Pharmacology.

[28] Mandal MD, Mandal S (2011). Honey: its medicinal property and antibacterial activity. Asian Pac J Trop Biomed.

[29] White JW, Subers MH (1963). Studies on honey inhibine. 2. A chemical assay. J Apic Res.

[30] Blair SE, Cokcetin NN, Harry EJ, Carter DA (2009). The unusual antibacterial activity of medical-grade *Leptospermum* honey: antibacterial spectrum, resistance and transcriptome analysis. Eur J Clin Microbiol Infect Dis.

[31] Irish J, Blair S, Carter DA (2011). The antibacterial activity of honey derived from Australian flora. PLoS One.

[32] Kwakman PHS, de Boer L, Ruyter-Spira CP, Creemers-Molenaar T, Helsper JPFG (2011). Medical-grade honey enriched with antimicrobial peptides has enhanced activity against antibiotic-resistant pathogens. Eur J Clin Microbiol Infect Dis.

[33] Morgulis S (1930). Studies of the inactivation of catalase. J Biol Chem.

[34] Mahmoud A, Owayss A (2006). A modified method to determine hydrogen peroxide activity as a quality criterion of bee honey. Ann Agric Sci.

[35] Molan PC (1992). The antibacterial activity of honey. 2. variation in the potency of the antibacterial activity. Bee World.

[36] Alaux C, Ducloz F, Crauser D, Le Conte Y (2010). Diet effects on honeybee immunocompetence. Biol Lett.

[37] Basualdo C, Sgroy V, Finola MS, Marioli JM (2007). Comparison of the antibacterial activity of honey from different provenance against bacteria usually isolated from skin wounds. Vet Microbiol.

[38] Huidobro JF, Sánchez MP, Muniategui S, Sancho MT (2005). Precise method for the measurement of catalase activity in honey. J AOAC Int.

[39] Schepartz AI (1966). Honey catalase: occurrence and some kinetic properties. J Apic Res.

